# Gene/protein name recognition based on support vector machine using dictionary as features

**DOI:** 10.1186/1471-2105-6-S1-S8

**Published:** 2005-05-24

**Authors:** Tomohiro Mitsumori, Sevrani Fation, Masaki Murata, Kouichi Doi, Hirohumi Doi

**Affiliations:** 1 Graduate School of Information Science, Nara Institute of Science and Technology, 8916-5, Takayama-cho, Ikoma-shi, Nara, 630-0101, Japan; 2 National Institute of Information and Communications Technology, 3-5 Hikaridai, Seika-cho, Soraku-gun, Kyoto 619-0289, Japan

## Abstract

**Background:**

Automated information extraction from biomedical literature is important because a vast amount of biomedical literature has been published. Recognition of the biomedical named entities is the first step in information extraction. We developed an automated recognition system based on the SVM algorithm and evaluated it in Task 1.A of BioCreAtIvE, a competition for automated gene/protein name recognition.

**Results:**

In the work presented here, our recognition system uses the feature set of the word, the part-of-speech (POS), the orthography, the prefix, the suffix, and the preceding class. We call these features "internal resource features", i.e., features that can be found in the training data. Additionally, we consider the features of matching against dictionaries to be external resource features. We investigated and evaluated the effect of these features as well as the effect of tuning the parameters of the SVM algorithm. We found that the dictionary matching features contributed slightly to the improvement in the performance of the f-score. We attribute this to the possibility that the dictionary matching features might overlap with other features in the current multiple feature setting.

**Conclusion:**

During SVM learning, each feature alone had a marginally positive effect on system performance. This supports the fact that the SVM algorithm is robust on the high dimensionality of the feature vector space and means that feature selection is not required.

## Background

There is a growing interest in genome research, and a vast amount of biomedical literature related to it has been published. Collecting and maintaining various databases of this information in computer accessible format require automatically extracting information. Various automated information extraction systems for biomedical literature have been reported. Ono et al. [1] and Blaschke et al. [2] demonstrated the automated extraction of protein-protein interactions (PPIs) from biomedical literature. They identified key words that express these interactions, and demonstrated automated extraction based on these key words and some heuristic rules. Temkin et al. [3] demonstrated the automated extraction of PPIs using a context-free grammar. In each of these studies, protein name recognition was the first step. Next, protein name dictionaries were constructed. Finally, protein name recognition was performed based on pattern matching using the dictionaries. Recognition performance affected the results of PPIs extraction. Fukuda et al. [4] and Frenzén et al. [5] developed automated recognition systems based on hand-crafted rules. They identified key terms for recognizing protein names, which they termed "core terms" (e.g. capital letters and special symbols) and "feature terms" (e.g. "protein" and "receptor"). Their systems recognize protein names based on these key terms and some hand-crafted rules.

Collier et al. [6] and Shen et al. [7] investigated the automated recognition of biomedical named entities based on the hidden Markov model. Kazama et al. [8], Lee et al. [9], and Takeuchi et al. [10] investigated automated recognition based on a support vector machine (SVM). Features for recognizing named entities were proposed in these investigations, e.g. word, part-of-speech (POS), and orthography.

Task 1.A of BioCreAtIvE was a competition involving automated gene/protein name recognition. The system described here was developed for that competition. It uses the SVM algorithm as a learning method for gene/protein name recognition. This algorithm has achieved good performance in many classification tasks, and we have previously showed that it performs well for protein name recognition [11]. Gazetteers have often been used for the named entity recognition task on newswire corpora. However, as Tsuruoka et al. [12] reported, dictionary pattern matching can result in low recall in a biomedical domain due to spelling variations. We thus investigated and evaluated the performance of our system when using an additional feature of making partial dictionary pattern matches. The gene/protein name dictionaries were made by collecting gene and protein names from the SWISS-PROT [13] and the TrEMBL [13] databases. We used partial dictionary matching, and the matches found in the dictionary became features used by SVM. Here we report the performance of our system in the BioCreAtIvE competition, analyze its features, and discuss the effect of the parameters on SVM learning.

## Results

### System description

**Figure 1:**
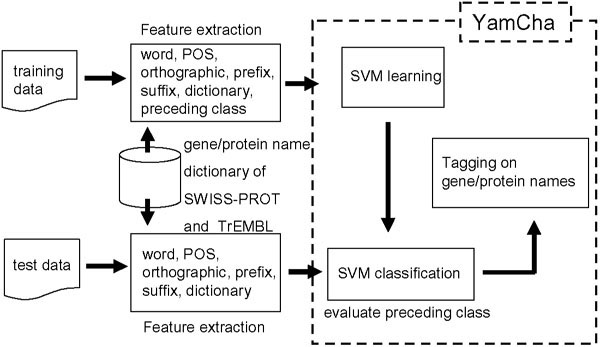
System concept.

The concept of our system is shown in Figure 1. We use SVM as the machine learning algorithm. The training data is a set of feature vectors with a binary value (+1 for a positive example and -1 for a negative example). The algorithm finds an optimal classification function that divides the positive and negative examples. We report on the features that we used in our system to recognize gene/protein names. We use the Yet Another Multipurpose Chunk Annotator, YamCha [14], which uses TinySVM  to bridge the gap between the results of feature extraction and the SVM.

### Training data and test data

Training data (7500 sentences) and development test data (2500 sentences) were prepared for system development in Task 1.A of the BioCreAtIvE competition. In both data sets, the gene/protein names were tagged with NEWGENE. Other tokens were tagged with POS tags. An example is the following: "translocation/NN of/IN the/DT NF-kappaB/NEWGENE transcription/NEWGENE factor/NEWGENE,/,", where NN is a noun or singular mass, IN is a preposition or subordinating conjunction, and DT is a determiner. This data is tokenized by BioCreAtIvE. We follow their definition. For example, "translocation", "of", "the", "NF-kappaB", "transcription", "factor" and "," are the tokens in above sample phrase.

### BIO representation

We used a BIO representation for chunking, using the following three tags.

• B: Current token is the beginning of a chunk.

• I: Current token is inside a chunk.

• O: Current token is outside of any chunk.

The resulting chunking representation of the above sample phrase is "translocation/O of/O the/O NF-kappaB/B transcription/I factor/I,/O".

### Feature extraction

We extracted the following features (see Table 1).

**Figure 2:**
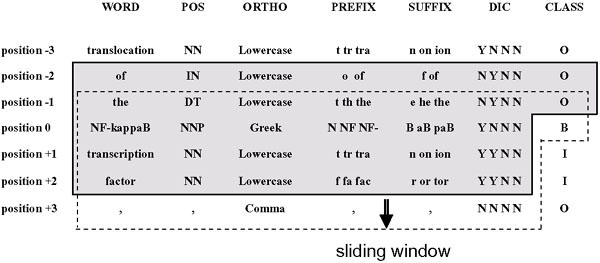
**Feature extraction example. **Feature extraction is shown using the sample phrase "...translocation of the NF-kappaB transcription factor...".

• Word: All words appearing in the training data.

• POS: Part of speech of the current token. We used the Brill tagger [15]. POS and NEWGENE were tagged in the training and test data for development. The tags were not shown in the test data for evaluation. We tagged using the Brill tagger in the training and two test data sets because we wanted to use POS as a feature.

• Orthography: Table 2 shows the orthographic features. If the token has more than one feature, then we used the feature listed first in Table 2 (left side comes before the right side in the table).

• Prefix: Uni-, bi-, and tri-grams (in letters) of the beginning letters of the current token.

• Suffix: Uni-, bi-, and tri-grams (in letters) of the ending letters of the current token.

• Dictionary matching: Matching gene/protein names dictionary entries against uni-, bi-, and tri-grams (in tokens) of words starting at the current token. For example, in Figure 2, the uni-gram (the current token) is "NF-kappaB", the bi-gram is "NF-kappaB transcription" and the tri-gram is "NF-kappaB transcription factor". There are four features: gene name dictionary match for the uni-gram (1), and protein name dictionary match for the uni-gram (2), bi-gram (3) and tri-gram (4). Each feature was represented as either Y (matching) or N (not matching). The dictionary was constructed based on the gene/protein names from the SWISS-PROT and TrEMBL databases. The SWISS-PROT database is a protein knowledge base including amino acid sequences and other properties currently known about the proteins. It is manually annotated. The TrEMBL database consists of computer-annotated entries derived from the translation of all coding sequences in the nucleotide sequence database. The sequences are not yet represented in the SWISS-PROT database. The TrEMBL database also contains protein sequences extracted from the literature and protein sequences submitted directly by the user community. We collected 96,195 protein names and 115,663 gene names from the SWISS-PROT database and 76,596 protein names and 31,414 gene names from the TrEMBL database. Two dictionaries were constructed, one from SWISS-PROT (GPD1) and the other from SWISS-PROT and TrEMBL (GPD2). When used for matching, each of these dictionaries is divided into 2 (sub-)dictionaries, one with the protein names and the other with the gene names. In our dictionary matching, we ignored the case and stop words, which are shown in Table 3. PubMed is a service of the National Library of Medicine that can be used to search for articles from over 15 million citations for biomedical articles. PubMed ignores the stop words in search queries.

• Preceding class: Class (i.e. B, I, or O) of the token(s) preceding the current token. The number of preceding tokens is dependent on the window size (described later on).

For example, the features of "NF-kappaB" in the sample phrase in Figure 2 are as follows. The word feature is NF-kappaB. The POS feature is NNP. The orthographic feature is Greek. The prefix features are N, NF, and NF-. The suffix features are B, aB, and paB. The value for uni-gram (NF-kappaB) matching with the protein name dictionary is Y. The value for bi-gram (NF-kappaB transcription) matching is N. The value for tri-gram (NF-kappaB transcription factor) matching is N. The value for uni-gram (NF-kappaB) matching with the gene name dictionary is N. The preceding class features are "O" for "the" (preceding first) and "O" for "of" (preceding second).

### Machine learning using YamCha

YamCha is a general purpose SVM-based chunker. YamCha takes in the training and test data and formats it for the SVM. The format of YamCha for a sample phrase is shown in Figure 2. The phrase is written vertically in the WORD column. The extracted features are shown in the following columns, e.g. the orthographic feature is shown in the ORTHO column. In the DIC column, the first three items show the results (Y or N) of the uni-, bi-, and tri-gram (in token) matching for the protein names in the dictionary. The last item shows the result of the uni-gram (in token) matching for the gene names in the dictionary. The CLASS column shows the class for each word, i.e., B, I, or O. Each feature is set apart by white space. The shaded area in Figure 2 shows the elements of the feature vectors for the current word, i.e. "NF-kappaB". The information from the two preceding and two following tokens is used for each vector. YamCha counts the number of features, and changes each feature into a unique positive integer. The feature vector transferred to the SVM by YamCha is in the form

+1 201:1 3148:1 4882:1 

-1 874:1 3652:1 6179:1 .

Each line shows one vector. A +1(-1) means a positive example (negative example). The positive integer on the left side of the colon is the unique number of each feature. A "1" on the right side of the colon shows that the vector includes the feature presented by the unique number.

We used three classes, i.e., B, I, and O. YamCha counted the number of classes appearing in the training data and directed the SVM to learn based on the situation. Three training sessions were done in a pair-wise fashion, i.e. (B vs. I), (B vs. O), and (I vs. O), and three hyperplanes were formed. In the test data, the optimal class of each token was the class that had the maximum value in the three hyperplane functions. Several parameters affect the number of support vectors.

• Dimension of polynomial kernel (natural number): We can use only a polynomial kernel in YamCha.

• Range of window (integer): The SVM can use the information on tokens surrounding the token of interest as illustrated in Figure 2.

• Method for a solving multi-class problem: We can use the pair-wise or one-vs.-rest method. In the latter, the B, I, and O classes are learned as (B vs. other), (I vs. other), and (O vs. other).

• Cost of constraint violation (floating number): There is a trade-off between the training error and the soft margin of the hyper plane.

### BioCreAtIvE Competition

The features and parameters we used in the BioCreAtIvE competition are shown in Tables 1 and 4. We tested three methods for the dictionary matching (1^st^, 2^nd^, and 3^rd ^runs).

• 1^st ^run: Exact pattern matching between GPD1 and words in the training and test data.

• 2^nd ^run: Regular expression pattern matching between GPD1 and words in the training and test data. We ignored non-alphabetical letters in the strings by using a regular expression in Perl. For example, "NF-kappa B" was represented as "NF\W*kappa\W*B". "\W" matches any character except a letter, numeric digit or "_". "*" indicates any number of such characters can be matched.

• 3^rd ^run: Exact pattern matching between GPD2 and words in the training and test data.

Our final result in the BioCreAtIvE competition is shown as case 1 in Table 5. The best "balanced f-score" was that for the 2^nd ^run. The differences between the three runs were less than 1%. The 3^rd ^run was the worst. The results without dictionary matching are shown as case 2 in Table 5. The score went up about 2.5% when dictionary matching was used.

### Analysis of recognition error

**Figure 3:**
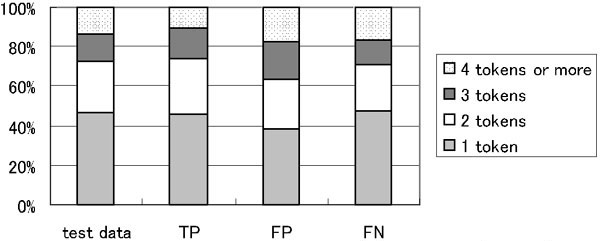
Percentage of n-grams of gene/protein names in test data for evaluation and TP, FP, and FN datasets.

Some gene/protein names are compound tokens. The average number of tokens per name was 2.131 tokens in the training data. In the first run, the average number of tokens per name were 1.998 (TP), 2.406 (FP), and 2.298 tokens (FN). Figure 3 shows the percentage of the number of tokens per gene/protein name that appeared in the test data for evaluation and the TP, FP, and FN data sets. The percentages of names over 4 or more tokens long in the FP and FN were higher than the one for TP, suggesting that recognizing longer names is more difficult than recognizing shorter ones.

Table 6 shows the number of overlapping gene/protein names between pairs of data sets (e.g. TP and FP). 76 names overlapped between the training data and FP of the test data, which was 8.1% of the names in the FP data. 50 names overlapped between the TP and FP of the test data, which was 5.3% of the names in the FP data. "TSST-1" and "PTH" are two example tokens that are sometimes marked as part of a gene/protein name in the evaluation test set and sometimes not. "TSST-1" is marked as part of a gene/protein name in test set sentence 11506, but not in sentence 10931. Similarly, "PTH" is marked as part of a gene/protein name in sentence 14477, but not in sentence 12212. The sentences are shown below with each token in the form *t*/*p*, where *t *is the token itself. *p *is 'NEWGENE' if *t *is part of a gene/protein name, otherwise, *p *gives the part-of-speech for *t*:

• @@11506 With/IN a/DT cutoff/NN level/NN for/IN TSST-1/NEWGENE of/IN ... were/VBD positive/JJ for/IN TSST-1/NEWGENE ./.

• @@10931 ... under/IN the/DT influence/NN of/IN TSST-1/NN ./.

• @@14477 ... the/DT secretion/NN of/IN PTH/NEWGENE and/CC CT/NEWGENE ...

• @@12212 ... and/CC intact/JJ PTH/NN (/(r/JJ =/SYM -0/CD ./CD 59/CD,/, p/NN </SYM 0/CD ./CD 05/CD)/SYM ./.

### Effects of tuning parameters and features

We analyzed the effects of the tuning parameters and features on system performance. In the analysis, we used the combined data set of the original training, development test and evaluation test. We performed a 10-fold cross validation using 90% of the combined data set as training data and the remainder as test data. This combined data set was also used in the analyses shown in Tables 8,9,10,11.

Table 7 shows the effects of tuning the parameters on precision, recall, and the balanced f-score. We define the parameters used in the 1^st ^run in the BioCreAtIvE competition as the "base" (see sub-section **BioCreAtIvE Competition **and Table 4). The balanced f-score decreased about 0.03 in the cases of deg(ree) = 1 and win(dow) = -1 to 1.] The other parameters did not have much effect on the results. We also carried out a Wilcoxon signed-ranks tests (two-sided) between the "base" and other cases. Statistical analysis was performed using commercial software (SPSS for Windows, version 11.0J, SPSS Inc.). Yeh [16] pointed out that "both precision and f-score are complicated nonlinear functions of random variables" and the randomization test was recommended. We used a Wilcoxon signed-ranks test with exact tests. A data sample for a test is the result of interest from one of the 10 trials in a 10-fold cross validation. Each sample was the difference between a trial's result for the case being compared and the corresponding result for the "base" case (a matched pair).

The p-values are shown in parentheses. We defined the null hypothesis as the case where there was no statistically significant difference between two values. The alternative hypothesis was defined as the opposite case. We set the statistical significance level at 0.05. The null hypotheses of the "deg.","win.-1+1", "cv = 0.01", recall of "win.-3+3" and precision of "one-vs.rest" cases were rejected. In other words, a statistically significant difference was seen in those cases.

The effects of each feature on our system are shown in Tables 8 and 9. The parameters are shown in Table 4. The definition of "base" is the same as in Table 7. In Table 8, we demonstrate the ability of SVM learning to be mostly unaffected when ignoring one of the features. The "-word" column in Table 8 shows the case in which the word feature was ignored. The other columns have a similar meaning. The suffix (preceding class) feature greatly affected recall (precision). The other features had little effect.

Table 9 showed the effect of adding the other features to the "base". In this table, the combination of the word and preceding class feature is defined as the "base". None of the features affected precision much, while all of them affected recall.

### Effect of dictionary matching methods

Table 10 shows the effect of each dictionary matching method. We used the features and parameters shown in Tables 1 and 4. A statistically significant difference is not seen. We also show the case in which the stop words were not ignored in the dictionary matching. A statistically significant difference was seen in this case, so stop words should be ignored in dictionary matching.

### Effect of POS tagger

As mentioned above, we used the Brill tagger [15] for POS tagging. The tagger was trained on newswire articles. Shen et al. [7] suggested that a POS tagger should be trained using this domain. They trained their tagger using the GENIA corpus [17]. They reported that using the POS feature greatly increased the f-score. We trained a tagger using 500 abstracts from GENIA corpus 3.02p. After the training, the accuracy of the POS tagging increased from 79.95 to 92.81% on the GENIA corpus.

Table 11 shows the effect of training on different corpora on the POS tagger. The "word+POS(A)+pc." column shows the case using the original POS tagger. The "word+POS(B)+pc." column shows the case using the POS tagger trained on the GENIA corpus. The word and preceding class features were also used in both cases. The f-score using GENIA trained POS tagger was worse than that using the original POS tagger. The "full(A)" and "full(B)" columns show the cases using the original and GENIA trained POS tagger, respectively. The difference between the "full" cases was small, but like with "word+pc.+POS" cases, instead of an expected increase, there was surprisingly a drop (in this case, not statistically significant) in the balanced f-score when switching from the original POS tagger to the GENIA trained POS tagger.

## Discussion

As described above, the system we developed for automated gene/protein name recognition uses the SVM algorithm and an SVM-based chunker, YamCha. YamCha performs pre-processing and set-up for the SVM. We evaluated our system in the BioCreAtIvE competition using various parameters for SVM learning, i.e. degree of the polynomial kernel, range of the window, cost of constraint violation, and method for solving multi-class problems. Among these parameters, the degree of the polynomial kernel (d = 1) and the range of the window (-1+1) had a significant effect. Takeuchi et al. [10] also used an SVM-based system to evaluate the effects of feature sets and found a range of -1+1 was better than -3+3, which is the opposite of our findings. The optimal window size is thus corpus dependent.

We also evaluated the effect of various features (both individually and combined): word, POS, orthography, prefix, suffix, dictionary matching, and preceding class features. In Table 8, one feature is removed at a time. As shown in the table, except for the suffix and preceding class features, removing any one feature did not have a large effect. In Table 9, the evaluations were performed by using each feature one at a time in addition to the word and preceding class features. As shown in the table, each feature had a significant effect when few other features were present. Combining this with the Table 8 results suggests that the features' effects usually overlapped with each other when the features were combined. Takeuchi et al. [10] reached to the same conclusion from their results.

As noted above, Tsuruoka et al. [12] reported that dictionary pattern matching can result in low recall in a biomedical domain due to spelling variations. As described above, we used uni-, bi-, and tri-gram (in tokens) matching for gene/protein name descriptions in the dictionary. The effect of dictionary matching feature was significant by itself. But when the features were combined, the effect was not so significant. Evaluation of different types of dictionary matching (Table 10) showed that the differences between the types were small.

We evaluated the effect of training a POS tagger with a corpus that is specific to the biomedical domain, as opposed to training the tagger on newswire. There was surprisingly a drop in the balanced f-score when switching from the original POS tagger to the GENIA trained POS tagger. One of the reasons may be that the GENIA corpus is smaller than the other training corpus.

## Conclusion

We participated in Task 1A of the BioCreAtIvE competition using the SVM algorithm. We evaluated the effect of several features on SVM learning. We also introduced information from external resources (gene/protein name databases) as a feature. While the effect of each feature was significant, these effects overlapped with each other when the features were combined. The best balanced f-score was the case where all features were used. This suggests that the effect of each feature was small when the features were combined. Kudo et al. [14] suggested that an SVM has a high generalization performance independent of the dimension of the feature vectors. Our results support their suggestion.

## Methods

The support vector machine (SVM), introduced by Vapnik [18], is a learning algorithm for solving two-class pattern recognition problems and is known for its good performance. SVM is used for many types of natural language processing (e.g. text classification and named entity recognition).

Each training data sample is labeled either a positive or negative example.

(**x**_1_, *y*_1_), ..., (**x**_*k*_, *y*_*k*_) **x**_*i*_∈ **R**^*n*^, *y*_*i *_∈ {+1, -1}.     (1)

**Figure 4:**
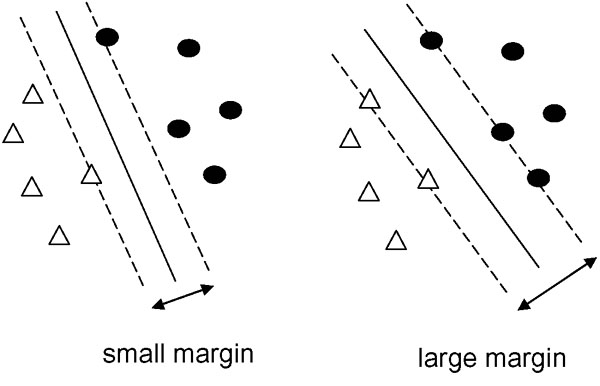
**Two possible hyper planes and margins between positive and negative examples. **White triangles are positive examples. Black dots are negative examples. A solid line shows a hyper plane. Two dashed lines are the boundaries between the positive and negative examples. An SVM finds the optimal hyperplane, which is the one with the maximum margin.

**x**_*i *_is the feature vector of the i-th sample and has a dimension of *n*. *k *is the total number of vectors. The *y*_*i *_is the class of the vector; it is either a positive example (+1) or negative example (-1). SVM separates the two types of examples with a hyperplane, as illustrated in Figure 4 (the solid line shows the plane). In this figure, triangles are positive examples and black dots are negative examples. The hyperplane is given by

(**w**·**x**) + *b *= 0 **w **∈ **R**^*n*^, *b *∈ **R**.     (2)

The two dashed lines in Figure 4 are the boundaries between the positive and negative examples. They are parallel to the hyperplane, and the distance between them is called the margin. In this figure, two possible separating patterns are shown: a small margin and a large margin. The two dashed lines and margin d are given by

(**w**·**x**) + *b *= ± 1, d = 2/||**w**||.     (3)

An SVM finds the values for the variables **w **and *b *which maximizes the margin for the training data.

These variable values minimize ||**w**|| under the constraint *y*_*i *_[(**w**·**x**) + *b*] ≥ 1.

In general, while the training data cannot be linearly separated, the non-linear boundary can be made linear using a kernel function *K*(**x**_*i*_, **x**_*j*_), which moves the training data to a high-dimensional space. Of the various types of kernels available, we used a d-th polynomial kernel:

*K*(**x**_*i*_, **x**_*j*_) = (**x**_*i*_·**x**_*j *_+ 1)^*d*^.     (4)

## Figures and Tables

**Table 1:** Features extracted.

Feature	Value
word	all words in the training data
orthography	capital, symbol, etc. (see Table 2)
prefix	1, 2, or 3 gram of the starting letters of a word
suffix	1, 2, or 3 gram of the ending letters of a word
part of speech	Brill tagger
preceding class	-2, -1
gene/protein name dictionary	protein names collected from SWISS-PROT and TrEMBL

**Table 2:** Orthographic features.

Feature	Example	Feature	Example
DigitNumber	15	CloseSquare	]
Greek	alpha	Colon	:
SingleCap	M	SemiColon	;
CapsAndDigits	I2	Percent	%
TwoCaps	RalGDS	OpenParen	(
LettersAndDigits	p52	CloseParen	)
InitCaps	Interleukin	Comma	,
LowCaps	kappaB	FullStop	.
Lowercase	kinases	Determiner	the
Hyphen	-	Conjunction	and
Backslash	/	Other	* + #
OpenSquare	[		

**Table 3:** Stop words defined by PubMed. Stop words were ignored during dictionary matching.

a	did	it	perhaps	these
about	do	its	quite	they
again	does	itself	rather	this
all	done	just	really	those
almost	due	kg	regarding	through
also	during	km	seem	thus
although	each	made	seen	to
always	either	mainly	several	upon
among	enough	make	should	use
an	especially	may	show	used
and	etc	mg	showed	using
another	for	might	shown	various
any	found	ml	shows	very
are	from	mm	significantly	was
as	further	most	since	we
at	had	mostly	so	were
be	has	must	some	what
because	have	nearly	such	when
been	having	neither	than	which
before	here	no	that	while
being	how	nor	the	with
between	however	obtained	their	within
both	i	of	theirs	without
but	if	often	them	would
by	in	on	then	
can	into	our	there	
could	is	overall	therefore	

**Table 4:** Parameters of YamCha. These parameters affect the support vectors in SVM learning.

Parameter	Value
kernel	polynomial
degree of kernel	2
direction of parsing	forward
window size	5 words (position -2, -1, 0, +1, +2)
cost of constraint violation	1.0
multi-class	pair-wise

**Table 5:** Evaluation results. Case 1 is using the dictionary feature. Case 2 is not using the dictionary feature. "TP", "FP", and "FN" are the numbers of true-positives, false-positives, and false-negatives.

	run	Precision	Recall	Balanced f-score	TP	FP	FN
	1^st^	0.8245	0.7416	0.7809	4412	939	1537
case 1	2^nd^	0.8230	0.7433	0.7811	4422	951	1527
	3^rd^	0.8225	0.7408	0.7795	4407	951	1542

case 2	without dictionary matching	0.8122	0.7075	0.7562	4209	973	1740

**Table 6:** Overlap of gene/protein names between any two data items. The results are for the first run. The numbers are the overlap between the row/column items.

	TP	FP	FN
training	1290	76	240
TP	-	50	194

**Table 7:** Effects of tuning parameters on system performance. The "base" parameters are shown in Table 4. The "deg." means the degree of the polynomial kernel, the "win." is the window range, and "cv" is the cost of constraint violation. The cost is the trade-off between the training error and margin. The "one-vs.-rest" method was used for solving multi-class problems. The parenthesized values are the p-values. The values in bold font have a statistically significant difference from the base value. A difference is labeled statistically significant when the p-value is less than 0.05 on the Wilcoxon signed-ranks sum test (two-sided).

	base	deg. = 1	deg. = 3	win. -1+1	win. -3+3	cv = 0.01	cv = 10	one-vs.-rest
Precision	0.8189	**0.7692 **(0.002)	**0.8266 **(0.014)	**0.7761 **(0.002)	0.8258 (0.064)	**0.8227 **(0.002)	0.8190 (0.750)	**0.8227 **(0.006)
Recall	0.7661	**0.7525 **(0.004)	**0.7395 **(0.002)	**0.7524 **(0.010)	**0.7546 **(0.002)	**0.7686 **(0.008)	0.7662 (1.000)	0.7655 (0.570)
Balanced f-score	0.7916	**0.7607 **(0.002)	**0.7806 **(0.006)	**0.7640 **(0.002)	0.7885 (0.232)	**0.7946 **(0.004)	0.7916 (0.500)	0.7930 (0.232)

**Table 8:** Effect of each feature on system performance I. The first column shows the values when all features were used in the SVM learning (word, POS, orthography (orth.), prefix (pre.), suffix (suf.), dictionary matching (dic.), and preceding class (pc.)). The other columns show the values when a feature was ignored in the learning. The parenthesized values are the p-values. The values in bold have a statistically significant difference from the base value. A difference is labeled statistically significant when the p-value is less than 0.05 on the Wilcoxon signed-ranks sum test (two-sided).

	base	-word	-pos.	-orth.	-pre.	-suf.	-dic.	-pc.
Precision	0.8189	**0.8076 **(0.002)	**0.8239 **(0.037)	0.8210 (0.375)	**0.8051 **(0.002)	**0.8000 **(0.002)	0.8143 (0.105)	**0.7009 **(0.002)
Recall	0.7661	**0.7619 **(0.008)	0.7658 (0.945)	**0.7590 **(0.020)	**0.7574 **(0.004)	**0.7233 **(0.002)	**0.7478 **(0.002)	**0.7508 **(0.002)
Balanced f-score	0.7916	**0.7840 **(0.002)	0.7937 (0.131)	0.7887 (0.160)	**0.7805 **(0.002)	**0.7597 **(0.002)	**0.7796 **(0.004)	**0.7250 **(0.002)

**Table 9:** Effect of each feature on system performance II. The first column shows the values when only the word and preceding class features were used in the SVM learning. The other columns shows the values when the word and preceding class features plus one other feature were used in the learning. The parenthesized values are p-values. The values in bold have a statistically significant difference from the base value. A difference is labeled statistically significant when the p-value is less than 0.05 on the Wilcoxon signed-ranks sum test (two-sided).

	word+pc. (base)	word+pc. +POS	word+pc. +orth.	word+pc. +pre.	word+pc. +suf.	word+pc. +dic.
Precision	0.8000	**0.7813 **(0.004)	**0.7886 **(0.014)	**0.7867 **(0.020)	0.8014 (0.770)	0.7964 (0.084)
Recall	0.5509	**0.6423 **(0.002)	**0.6786 **(0.002)	**0.6374 **(0.002)	**0.7035 **(0.002)	**0.6410 **(0.002)
Balanced f-score	0.6524	**0.7118 **(0.002)	**0.7295 **(0.002)	**0.7041 **(0.002)	**0.7492 **(0.002)	**0.7102 **(0.002)

**Table 10:** Effect of dictionary matching. The results are for a 10-fold cross validation. The results in Table 5 were for the evaluation test data; cross validation was not used. "GPD1" means the results using GPD1 in dictionary matching. They correspond to the 1^st ^run in Table 5. "GPD1 with regexp." means the results using GPD1 with regular expressions in dictionary matching. They correspond to the 2^nd ^run in Table 5. "GPD2" means the results using GPD2 in dictionary matching. They correspond to the 3^rd ^run in Table 5. "GDP1-stop words" means the results when the stop words were not ignored in dictionary matching on the 1^st ^run. The parenthesized values are p-values. The values in bold have a statistically significant difference from the 1^st ^value. A difference is labeled statistically significant when the p-value is less than 0.05 on the Wilcoxon signed-ranks sum test (two-sided).

	GPD1	GPD1 with regexp.	GPD2	GPD1-stop words
Precision	0.8189	0.8199 (0.695)	0.8130 (0.131)	0.8099 (0.006)
Recall	0.7661	0.7668 (1.000)	0.7596 (0.160)	0.7600 (0.049)
Balanced f-score	0.7916	0.7924 (0.770)	0.7854 (0.105)	0.7841 (0.006)

**Table 11:** Effect of trained POS tagger on biomedical literature. POS(A) means the original, newswire trained POS tagger. POS(B) means the POS tagger with trained on GENIA corpus 3.02p. "full(A)" means using all features and the original, newswire trained POS tagger. "full(B)" means using all features and the POS tagger with trained on GENIA corpus 3.02p The parenthesized values are p-values. We compare two cases: "word+POS(A)+pc." vs. "word+POS(B)+pc." and "full(A)" vs. "full(B)". The values in bold have a statistically significant difference in the comparison. A difference is labeled statistically significant when the p-value is less than 0.05 on the Wilcoxon signed-ranks sum test (two-sided).

	word+POS(A)+pc.		word+POS(B)+pc	full(A)		full(B)
Precision	0.7813		0.7867	0.8189		0.8177
	(0.105)			(0.557)		
Recall	**0.6423**		**0.6147**	0.7661		0.7640
	(0.002)			(0.322)		
Balanced f-score	**0.7118**		**0.6900**	0.7916		0.7899
	(0.002)			(0.432)		
